# Free superficial circumflex iliac artery perforator flap with a single-pedicle bilobed design for pediatric multi-digit defect reconstruction

**DOI:** 10.1186/s13018-020-01733-3

**Published:** 2020-06-11

**Authors:** Zhangcan Li, Dawei Zheng, Jian Zheng, Weiya Qi, Qiang Qi, Yunyun Liu

**Affiliations:** 1 Department of Hand Microsurgery, Xuzhou Renci Hospital, No. 11 Yangshan Road, Xuzhou, 221004 Jiangsu China; 2 Department of Gynecology, Xuzhou Maternal & Child Health Care Hospital, Xuzhou, 221009 Jiangsu China

**Keywords:** Iliac artery, Perforator flap, Pediatrics, Reconstructive surgical procedures, Finger injuries

## Abstract

**Objectives:**

This paper describes imaging and anatomical features, in order to assess the feasibility of superficial circumflex iliac artery perforator (SCIP) flap with a single-pedicle bilobed design for multi-digit skin and soft tissue reconstruction in pediatric patients.

**Methods:**

A total of 7 pediatric patients who were being treated with free single-pedicle bilobed SCIP flap reconstruction for multi-digit defects were included in this study. The details of the clinical features were collected, and the following were successively analyzed: the preoperative computed tomographic angiography (CTA) and color Doppler sonography (CDS) examinations for flap design, the intraoperative anatomy for perforator vessel, defect reconstruction and interphalangeal range of motion (ROM) and tactile sense, pain sense, and two-point discrimination recovery results.

**Results:**

CTA and CDS performed preoperatively could accurately and rapidly identify the position, location and course of the superficial circumflex iliac artery perforator. All wounds healed by the first follow-up and no complications occurred at the follow-up visit. All flaps survived, the patients achieved proximal interphalangeal joint (PIP) ranges of motions (ROM) from 80 to 100° and distal interphalangeal joint (DIP) ROM from 65 to 80°. The tactile sense and pain sense recovered, and average of the two-point discrimination scores was 9.3 mm (range 7–12 mm). The donor area was primarily sutured with a tidy scar in the underwear region.

**Conclusion:**

CTA and CDS performed preoperatively are accurate and intuitive methods for assessing the location and course of SCIP. The SCIP flap is suitable for pediatric patients due to its small vessel caliber, specific functional and esthetic benefits. It can be designed in a lobulated fashion in order to repair two or more wounds during one surgery. We suggest that the free single-pedicle bilobed SCIP flap should be considered a good option choice for multi-digit defect reconstruction in pediatric patients in the clinic.

## Introduction

The hands play a very important role in daily life and routine work; therefore, the finger and dorsum of the hand are most frequently subjected to soft tissue injuries of the skin [1, 2]. Children are naturally active and lack safety awareness, coupled with parents’ neglect of care, hand injuries are relatively common in early life [3].

Children’s fingers are thinner than adults’ fingers, and the diameters of blood vessels are also thinner, while the vessels of traditional flaps are larger, which makes vascular anastomosis more difficult. If multiple finger injuries occur, the traditional surgical method often involves the placement of an abdominal pedicled flap to artificially form combined fingers; then, the second stage of the operation is performed for pedicle division and finger splitting [4, 5].

Nevertheless, because of the long passive position, there are several limitations of the method, such as that it can result in joint stiffness, discomfort position and unexpected flap avulsion.

In recent years, with the continuous improvements in microsurgery technology, the free superficial circumflex iliac artery perforator(SCIP) flap, which evolved from the traditional groin flap, has achieved many outstanding advantages, such as its softness, flexibility, limited damage to the donor area, minimal donor site morbidity, and scar concealment [6]. At present, this flap is gradually applied for the repair of various skin and soft tissue defects in different sites, such as the head and neck, external auditory canal, limbs, perineum and penis, and relevant research has been reported in succession [6–10]. The SCIP flap is a suitable candidate owing to its unique characteristics, such as its thinness and pliability, the small caliber of the vessels, and the design flexibility; it can also reduce operation difficulty resulting from nonconformity of the vessel caliber. The flap can be designed with a lobulated appearance in order to repair two or more wounds simultaneously by anastomosing one group of blood vessels [11].

To our knowledge, there is no previous report of children’s multiple finger reconstruction with a single-pedicle bilobed SCIP flap. We report the application of free single-pedicle bilobed SCIP flap for skin and soft tissue reconstruction of children’s multiple finger defects which were successfully treated in our department in recent years. In our study, the fingers achieved a satisfactory appearance as well as motor function and protective sensation recovery, and the donor area was primarily sutured with the scars hidden. Based on our results, we report that the single-pedicle bilobed SCIP flap is an effective and optional choice for multi-digit defect reconstruction in pediatric patients.

## Materials and methods

### Clinical data

The clinical data of the patients who underwent the reconstruction of finger defect with a free superficial circumflex iliac artery single-pedicle bilobed SCIP flap from October 2015 to October 2018 in Xuzhou Renci Hospital was collected and presented as follows (Table 1). The parents of all children agreed and signed the informed consent in this study. Ethics approval was granted by Institutional Clinical Research Supervision Committee.

**Table 1:** Basic information, defect site, approximate defect area, and flap type of patients

Sex	Age (year)	Site	Defect area (cm)	Design	Donor site
M	11	Index-middle fingers	2.5 × 3.0–2.3 × 2.6	Single-pedicle bilobed	Primary closure
M	8	Thumb-index fingers	2.5 × 3.5–1.8 × 2.7	Single-pedicle bilobed	Primary closure
F	6	Middle-ring fingers	1.8 × 2.8–1.6 × 1.8	Single-pedicle bilobed	Primary closure
M	8	Index-middle fingers	2.1 × 3.3–1.8 × 2.3	Single-pedicle bilobed	Primary closure
M	4	Index-middle fingers	1.8 × 2.5–2 × 3.5	Single-pedicle bilobed	Primary closure
M	5	Middle-ring fingers	2.0 × 2.3–2.5 × 2	Single-pedicle bilobed	Primary closure
M	6	Ring-little fingers	1.7 × 2.5–1.8 × 2.5	Single-pedicle bilobed	Primary closure

Six men and one female pediatric patient were included in our study. In these 7 patients, the mean age of the patients was 6.85 years old (range from 4 to 11 years); defect cause was hank injury (3), crush injury (2), triangle belt injury (2); defect site was index-middle fingers (3), thumb-index fingers (1), middle-ring fingers (2), ring-little fingers (1); defect areas (one finger) range from 1.6 × 1.8 cm to 2.5 × 3.5 cm. Detailed information is shown in Table 1.

### Preoperative imageological examination and body surface marking for flap design

Given to the diversified anatomical variations of SCIP vessels mentioned in previous literature, preoperative imageological examination plays a crucial part in performing a successful operation. Preoperative color Doppler sonography (CDS) mapping and computed tomographic angiography (CTA) combined with three-dimensional (3D) volume-rendered reconstruction were performed to identify the location, course, perforator branch of SCIA in great details and then the body surface projection line was marked. All those were prepared to design appropriate flap for finger reconstruction.

### Surgical procedure

The patient was placed in a supine position under general anesthesia, and the edge of the defect was trimmed after thorough debridement of the necrotic skin and soft tissue of the fingers in order to identify the available arteriovenous vessels for anastomosis under a tenfold microscope.

The bilobed flap was then harvested according to the preoperative marker line drawn on the body. The flap was 10% larger than the defect area to avoid the flap shrink after harvest. The first incision was made along the marker line through the subcutaneous tissue and the surface of the tendinous membrane of the external oblique to cautiously expose the SCIP. All small vessels are dissected and ligated or cauterized. The superficial fascia was opened and blunt separation was continued in order to identify the SCIP vessels. Afterwards, careful pedicle dissection was performed to harvest the flap after the excision of subcutaneous tissues and arteriovenous vessels for anastomosis under a tenfold microscope. All the surgical operations were performed by the same surgeons from the first author’s team.

### Results

CTA and CDS are accurate and rapid means to identify the location and course of SCIP. All flaps survived uneventfully, and no complications were observed at the 8-to-14-month follow-up postoperatively except for one child with scar contracture of the interphalangeal joint. Finger function recovered well after release surgery and postoperative systematic rehabilitation training. All flaps survived, the donor area was primarily sutured and had a well-concealed scar. All flaps survived, the patients achieved PIP ROM from 80 to 100° and DIP ROM from 65 to 80°. And the tactile sense and pain sense recovered, and average of the two-point discrimination scores was 9.3 mm (range 7–12 mm).

### Classic case presentation

**Fig. 1:**
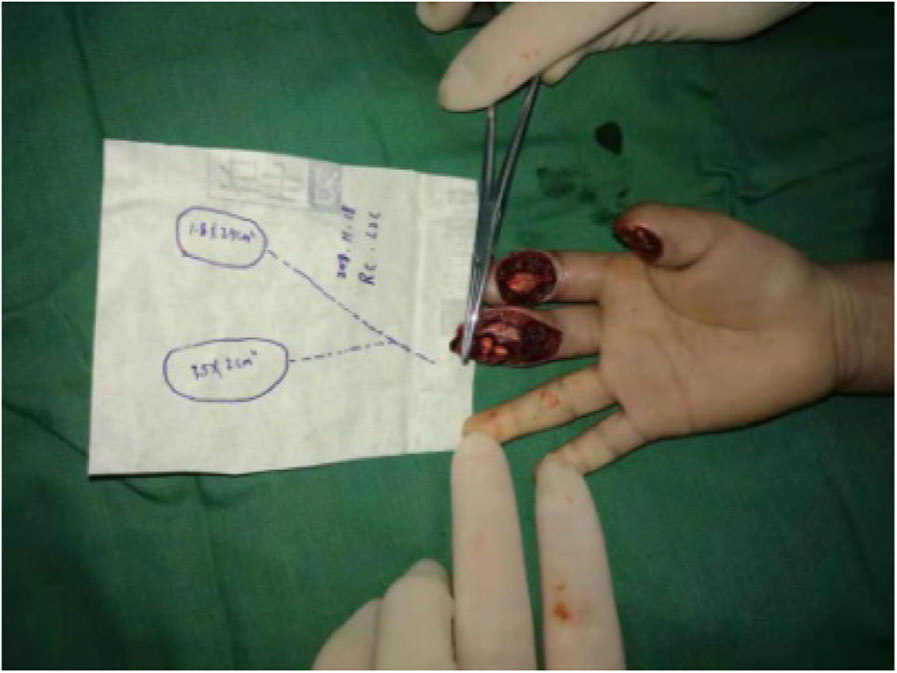
Preoperative view of right index and middle fingers

**Fig. 2:**
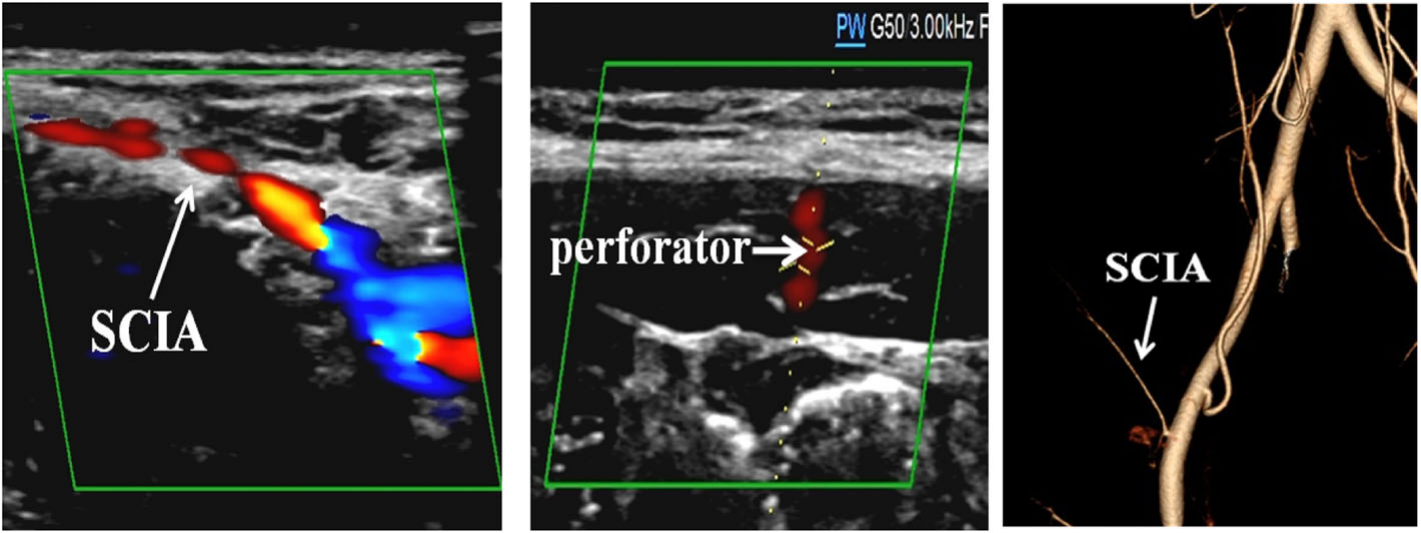
Preoperative CTA and CDS examinations

**Fig. 3:**
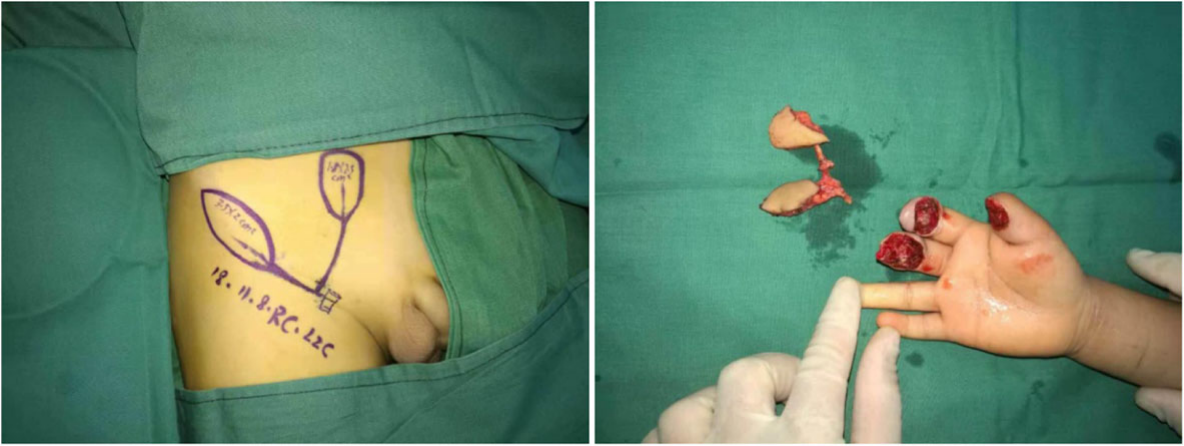
Flap marking and harvesting of the single-pedicle bilobed flap

**Fig. 4:**
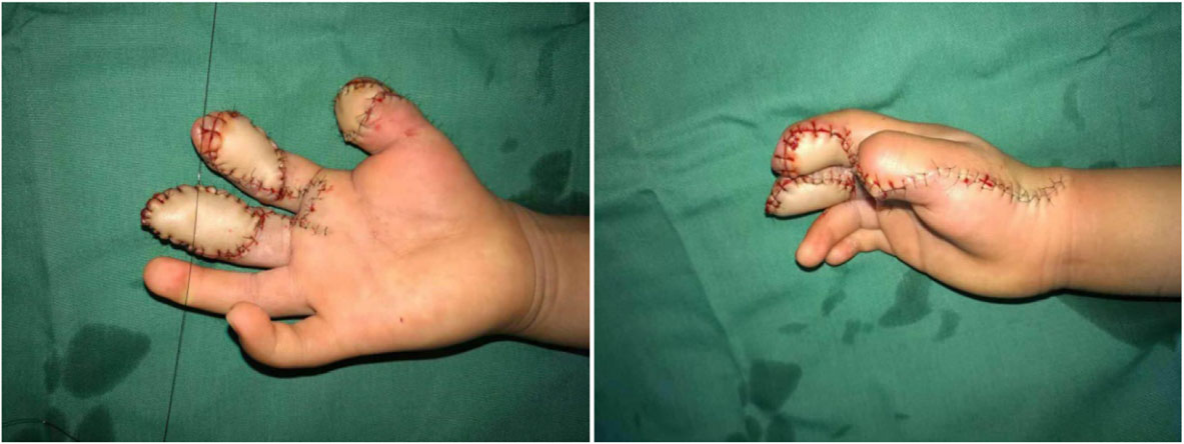
Flap reconstruction

**Fig. 5:**
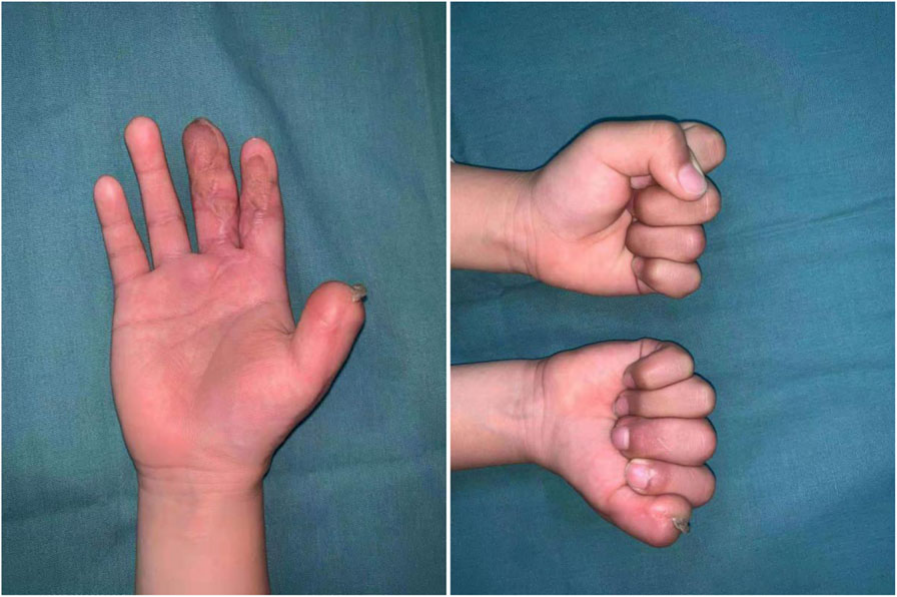
Postoperative result at 14-month follow-up visit

A 4-year-old child sustained a crushing injury to his right index and middle fingers, resulting in soft tissue defects with bone and tendon exposure (Fig. 1). A single-pedicle bilobed SCIP flap including one lobe of approximately 1.8 × 2.5 cm in size and another of approximately 3.5 × 2 cm in size was designed after CDS mapping and CTA examination (Figs. 2 and 3). The flap healed well with no complications, and the patient was discharged home on day 14 after reconstructive surgery (Fig. 4). The PIP ROM is 90° and DIP ROM is 65°, the tactile sense and pain sense recovered well, and the two-point discrimination scores was 8 mm. His parents remained contented with the interphalangeal ranges of motions and esthetic effect at the 14-month follow-up visit (Fig. 5).

## Discussion

The hand has been regarded as one of the most crucial organs for daily activities; thus, hand injuries are very common in the clinic. With the development of industrialization and with the presence of frequent accidents such as traffic accidents, hand injuries are currently increasing daily in society. Such injuries often result in the loss and necrosis of skin and soft tissue to different extents and are often accompanied by tendon exposure, nerve injury, and bone defects. Hand reconstruction is challenging due to the unique functions and appearance of the hand; above all, skin reconstruction in order to restore the function of the hand is always difficult in the clinic [2].

Due to the unique functions and appearance of the hand, to restore the glabrous skin of the radial digits is critical. An ideal tissue replacement should be antifriction, glabrous and with similar texture and plump subcutaneous tissue. It has been reported that kinds of flaps have been successfully applied in clinic: digital artery island flap [12], free fibula side flap [13], and thenar flap [14], for example. But, these flaps have some limitations, such as, skin grafting for donor area, postoperative scar, long operation time, and last but not least, the flaps could not be designed too large; they are more suitable for single digit with smaller defect area.

Since abdominal flaps were first reported by McGregor and Jackson in 1972, they have gradually become ideal workhorse flaps for skin and soft tissue replacement in the hand. Abdominal flaps have many advantages, such as their consistent vessel anatomy, flexible design, harvesting simplicity, rapid postoperative recovery, and limited complications and wound infections [15]. However, in addition to their advantages, these flaps also have limitations as well as disadvantages because if multiple finger injuries occur, the traditional surgical procedure used is often to artificially form syndactylia using an abdominal pedicled flap; this involves a secondary surgery for cutting the vascular pedicle and separating the fingers. Because of the long-term passive position, joint stiffness, discomfort position, and unexpected flap avulsion often occur [16, 17].

For decades, with the continuous development of microsurgery techniques and tools, supermicrosurgery, a technique involving the dissection and anastomosis of small vessels ranging from 0.3 to 0.8 mm in size, has revolutionized the field of vessel reconstruction, allowing the possibility of perforator flaps with thinner calibers [17]. The concept of a perforator flap was first presented by Koshim and Soeda in 1989, and this flap roughly experienced three substantial processes of development: pedicle flap-free flap-perforator flap [17, 18].

The perforator flap is a kind of flap that survives due to the blood supply from perforator vessels with small diameters (approximately 0.5 mm). Unlike other traditional flaps, the perforator flap does not depend on muscle or deep fascia and reduces the morbidity of the donor area to the minimum. Therefore, the use of this flap is an inevitable trend in the field of hand trauma and represents an opportunity for microsurgery to solve complex reconstructive problems. In recent years, various types of perforator flaps have been reported, such as the paraumbilical, anterolateral thigh, lateral thoracic, superficial circumflex iliac, and gluteal artery perforator flaps.

In 2004, Koshima et al. first established the concept of the SCIP flap, constituting the dissection of a groin flap based on the superficial circumflex iliac artery (SCIA) [19]. The SCIP flap is pliable, thin, and reliable and is one of the most demanding flaps with long vascular pedicles. This flap has many advantages by not only obtaining an esthetic effect and functional outcome but also minimizing donor site morbidity with concealment of the scar, reducing the surgical time, and achieving one-stage surgical reconstruction.

The SCIP flap is one of the most advantageous flaps because it enables the reconstruction of multiple lesions with one source vessel. It can be designed with a lobulated appearance in order to repair two or more wounds simultaneously by anastomosing one group of blood vessels.

In recent years, with the development of perforator flaps, many studies have reported that these flaps can be successfully applied in many fields. The SCIP flap was widely used since it was first described in 2004 by Koshima et al.; soon afterwards, Lita and his colleagues demonstrated success of the SCIP flap in reconstructive of head and neck defects [9]. It has been reported in many department listed as follows: reconstructions of hand, breast, lower extremity, penis, limbs [20], urethral reconstruction [21], oral maxillofacial reconstruction [22], and so on.

The SCIP flap plays an important role in clinical microsurgery due to its remarkable esthetic and functional merits. However, it also has some limitations; perforator flap surgery is relatively complicated mainly because of the short arterial pedicle, arterial anatomical variability and tiny caliber of the vessels. Due to these difficulties, the performer should be experienced instead of microsurgically novice.

Meanwhile, preoperative imageological examination and anatomical assessment of vascular mapping were important for the successful flap transfer. Diversified anatomical variations of dominant vasculature in the groin area especially SCIA have been reported in previous study [23, 24]. So preoperative CDS mapping and CTA combined with three-dimensional volume-rendered reconstruction were performed to identify the location, the whole vessel stream, vascular trunk, and side branch of SCIA. Following the tiny branches, the main trunk of the SCIA was detected, and then the projection lines were marked on body surface with gentian violet pen.

The SCIP flap is crucial for hand reconstruction not only in adult patients but also in pediatric patients. Previous relevant studies have mainly focused on adults, and few studies have reported the use of perforator flaps in pediatric patients. To be certain, vascular anastomosis in pediatric patients certainly poses a unique clinical challenge.

Children’s fingers are smaller than adults’ fingers, while the vascular caliber of the traditional flap is wider, which makes the anastomotic process more laborious. The SCIP flap, with its small vessel caliber, flexibility, minimal donor site morbidity, and scar concealment, is highly suitable for pediatric patients. This flap can be designed with a lobulated appearance to repair two or more wounds in order to avoid the inconsistencies in caliber and the risk of a second-stage operation. To our knowledge, the relevant study of pediatric patients is not common; this study is the first to conduct a free single-pedicle bilobed SCIP flap for multi-digit defect reconstruction in pediatric patients.

Seven pediatric patients who underwent the reconstruction of finger defect with free single-pedicle bilobed SCIP perforator flaps were collected, all flaps survived and with no complications except one child with scar contracture of interphalangeal joint, finger function recovered well after release surgery and postoperative systematic rehabilitation training, and the flaps achieved good interphalangeal ranges of motions as well as tactile sense, pain sense, and two-point discrimination recovery. The donor area is sutured primarily and with a well-concealed scar.

In spite of these advantages, our study is limited with the small sample size and lack of objective monitoring index. Also, the longstanding operation and difficulties in harvesting flap are apparent limitations. Further studies with larger sample should be conducted to consolidate this research and develop more practical variations.

## Conclusion

Altogether, CTA and CDS performed preoperatively are accurate and intuitive methods for assessing the location and course of SCIP. In our view, free single-pedicle bilobed SCIP flap can be considered a good option for multi-digit defect reconstruction of pediatric patients in clinic.
